# Crowded Nests: Parent–Adult Child Coresidence Transitions and Parental Mental Health Following the Great Recession

**DOI:** 10.1177/0022146519849113

**Published:** 2019-05-23

**Authors:** Jennifer Caputo

**Affiliations:** 1 Max Planck Institute for Demographic Research, Rostock, Germany

**Keywords:** intergenerational households, life course, mental health, parent–adult child coresidence, living arrangements, older adults

## Abstract

Although many studies have examined contemporary increases in parent–adult child coresidence, questions about what this demographic shift means for the well-being of parents remain. This article draws on insights from the life course perspective to investigate the relationship between parent–adult child coresidence and parental mental health among U.S. adults ages 50+, distinguishing between parents stably living with and without adult children and those who transitioned into or out of coresidence with an adult child. Based on analyses of the 2008 to 2012 waves of the Health and Retirement Study (*N* = 11,277), parents with a newly coresidential adult child experienced an increase in depressive symptoms relative to their peers without coresidential adult children. Further analyses suggest that transitions to coresidence that occurred in the southern United States or involved out-of-work children were particularly depressing for parents. These findings highlight the significance of evolving intergenerational living arrangements for the well-being of older adults.

The proportion of multigenerational households in the United States has grown over the last decades, particularly since the Great Recession of 2007 to 2009. While this demographic trend has been widely acknowledged, much of the scholarly and public attention to it focuses on young adults who have yet to leave or have returned to a parental home. However, increases in parent–adult child coresidence have affected parents and children of all ages and encompass a variety of situations (Courtin and Avendano 2016; Mykyta and Macartney 2011; Rappaport 2015). Although scholars have begun to assess how these arrangements are related to the well-being of the younger generation (Caputo 2018; Copp et al. 2017; Stone, Berrington, and Falkingham 2014), questions about what living with adult children means for contemporary aging parents remain, including whether the mental health of those who transition to, transition from, or have been consistently coresiding with an adult child differs from that of parents stably living without adult children. To the extent that transitions to coresidence with adult children violate expectations or introduce emotional or socioeconomic stressors, they may increase parental distress relative to other living arrangements. However, the mental health implications of coresidence transitions are likely to be contingent on other specific contexts, including whether the move is to the parent’s own or the child’s home, whose needs the move fulfills, and the characteristics of both parties.

This article uses panel data from the Health and Retirement Study (HRS) to assess the relationship between parent–adult child coresidence statuses between 2008 and 2010—including stable residential independence, stable coresidence with an adult child, transitions from living with an adult child, and transitions to living with an adult child—and parental depressive symptoms in 2012 among U.S. adults ages 50 and older. The analyses also explore whether the effects of these coresidential transitions depend on the parental characteristics and geographic contexts that predict them (Aquilino 1990; Smits, van Gaalen, and Mulder 2010; Vespa 2017). Then, focusing on parents with newly coresidential children, they assess whether the direction of the move, characteristics of return coresidential children, and reported beneficiary of the move are related to depression. To help account for the selection of parents who are on declining health trajectories into coresidence with adult children (Seltzer and Friedman 2014; Ward, Logan, and Spitze 1992), analyses include baseline health and make use of inverse-probability weights. This study draws on insights from the life course perspective highlighting the importance of the sociohistorical contexts surrounding and timing of transitions and the interdependent and linked nature of lives (Bengston and Allen 1993; Elder 1994; Elder, Johnson, and Crosnoe 2003). The findings are timely because this demographic trend shows no sign of reversing, suggesting that increasing proportions of adults can expect to find themselves living under the same roof as an adult child at some point in later life.

## Background

Multigenerational living arrangements declined steadily throughout much of the twentieth century in the United States (Bianchi and Casper 2000; Ruggles 2007). While 21% of the population resided in a household that included more than one adult generation in 1950, by 1980 that proportion had shrunk to 12% (Cohn and Passel 2016). The share has experienced regrowth since, passing 19% of the population in 2014 (Cohn and Passel 2016). Much of this recent increase is attributable to young adult children living in their parents’ homes—as do now nearly a third of those ages 18 to 34 (Fry 2016)—and a significant body of research describes and assesses this trend (e.g., Copp et al. 2017; Fry 2017; Houle and Warner 2017; Newman 2012; Sandberg-Thoma, Snyder, and Jang 2015; Sassler et al. 2008; Stone et al. 2014). However, coresidence with parents is also increasing among older adult children. The proportion of adults ages 40 to 44 living with their parents more than doubled between 1980 and 2013, from under 3% to over 7% (Rappaport 2015). And naturally, there are accompanying changes in the living arrangements of the older generation (Cohn and Passel 2016; Kahn, Goldscheider, and García-Manglano 2013). By 2010, more than 11% of adults ages 45 to 64 and 18% of adults 65 and older lived with an adult child or grandchild (Kahn et al. 2013). The increase in multigenerational coresidence thus has implications for growing swaths of the adult population of all ages.

Changes in the economy, especially since the Great Recession, are often cited as the main driver of increased multigenerational coresidence (Bell and Blanchflower 2011; Kahn et al. 2013). Indeed, research indicates that economic setbacks and the housing crisis pushed growing proportions of struggling younger generations into their parents’ homes (Fry 2017; Furstenberg 2010; Kahn et al. 2013; Maroto 2017; Rappaport 2015). However, there are other important reasons for the growth in multigenerational households. Family demographers point out that because of extending life expectancies, different generations are “living longer together” (Bengtson 2001; Gilligan, Karraker, and Jasper 2018; Swartz 2009). As the period that individuals can expect to live alongside their family members grows, so will opportunities for them to coreside.

Despite widespread recognition of this demographic trend and interest in its implications, several questions about whether and how living with adult children affects the well-being of contemporary parents remain. Drawing on insights from the life course perspective, the next sections describe the specific gaps and hypotheses investigated in this study.

### Coresidence with Adult Children and Parental Mental Health

A number of previous studies have explored relationships between coresidence with adult children and parental well-being. Some suggest that living with adult children has primarily negative effects on parents’ mental health (Johar and Maruyama 2014; Pudrovska 2009; Tosi and Grundy 2018). Explanations for this pattern include that coresidential adult children can drain parents’ financial assets (Maroto 2017), reduce the quality of marital relationships (Davis, Kim, and Fingerman 2016), create parent–child conflicts (Ward and Spitze 2007), and be disappointing or stigmatizing (Newman 2012; Sassler et al. 2008). More generally, the empty-nest stage is positively anticipated by many parents, and a returning child may be an unwelcome disruption (Aquilino and Supple 1991; Barber 1989). However, many other studies indicate that living with adult children has *positive* effects for parents (Aranda 2015; Chen and Short 2008; Courtin and Avendano 2016; Grundy and Murphy 2017). Researchers explain these findings by noting that coresidential adult children can offer their aging parents critical sources of social, instrumental, and financial support (Courtin and Avendano 2016; Grundy and Murphy 2017). The life course perspective provides a useful framework for interpreting these apparently contradictory findings.

Existing research often overlooks the dynamic, temporal context of parent–adult child coresidence, focusing on current coresidential status but not considering change. As the life course perspective emphasizes, statuses are characterized by transitions and turning points that carry implications for well-being (Elder 1994; Elder et al. 2003). Reflecting this, research examining coresidence transitions among young adults suggests that while returns to a parental home increase distress, stably living with parents does not (Caputo 2018; Copp et al. 2017; Sandberg-Thoma et al. 2015; South and Lei 2015). The mental health of young people leaving a parental home also does not appear to differ from that of the stably independent. However, most studies focusing on parents’ well-being do not differentiate between new and longer-term coresidential arrangements or assess whether there are changes in mental health among parents experiencing the departure of a coresidential adult child (Chan et al. 2011; Chen and Short 2008; Courtin and Avendano 2016; Grundy and Murphy 2017). A recent exception finds that having new adult child coresidents predicts decreased parental well-being in Europe (Tosi and Grundy 2018) but also does not distinguish between parents stably with and without coresidential adult children. Consistent with the findings for young adults, it is possible that living with adult children is distressing only among parents recently experiencing a transition, for whom it represents the introduction of new stressors and/or a loss of independence.

The life course principle of time and place highlights that individuals’ lives unfold within particular sociohistorical contexts (Elder 1994; Elder et al. 2003). However, existing studies cover a range of time periods, during which parent–adult child coresidence resulted from different processes and carried different meanings. As noted, contemporary increases in multigenerational households are linked to growing economic strains, particularly those encountered by the younger generation (Cohn and Passel 2016; Fry 2017). For example, Kahn and colleagues (2013) found that between 1960 and 2010, children’s needs for financial support from their parents became increasingly salient predictors of multigenerational coresidence. If the upturn in coresidential arrangements is indeed being driven by child need over parental choice, they may be becoming more distressing for parents.

Additionally, most previous work is based on non-U.S. samples. However, there are broad cross-national differences in the history and meaning of intergenerational coresidence. Newman (2012) found that parental attitudes toward coresidential adult children ranged from fear and shame in Japan to acceptance and even enjoyment in Italy. The only study described using U.S. data showed that living with children over age 23 predicted increased depressive symptoms among mothers (but not fathers) who were 64 to 65 in 2004 (Pudrovska 2009). This finding stands in intriguing contrast to much of the work based on other nations, which tends to indicate that coresidence is positively related to parental well-being (Aranda 2015; Courtin and Avendano 2016; Grundy and Murphy 2017). It may be that living with adult children is distressing for parents in the United States, where economic independence is especially valued (Settersten, Ottusch, and Schneider 2015), but not some other national contexts.

Together, these insights support the following hypothesis about the relationship between parent–adult child coresidence transitions in the contemporary United States and parental mental health:

**Hypothesis 1:** Parents experiencing a transition to coresidence with an adult child will report an increase in depressive symptoms relative to parents stably without coresidential children, while depressive symptoms among parents stably with or experiencing the exit of an adult coresidential child will not differ from those of stably independent parents.

### Parental Characteristic and Geographic Variations

Life course scholars emphasize that norms and expectations surrounding events and transitions can be powerful, with the potential to impact well-being (Bengston and Allen 1993; Elder et al. 2003; Elder and Rockwell 1978). Research reveals clear patterns in the types of parents that tend to live with their adult children, many of which may reflect parents’ own needs for social or financial support. Specifically, parents who have lower incomes and levels of education, are widowed or not working, and are in poorer health are more likely to live with an adult child than their more economically well-off, socially engaged, and healthier peers (Choi 2003; Kahn et al. 2013; Mykyta and Macartney 2011; Seltzer and Friedman 2014; Smits et al. 2010). Reflecting persistent gender norms about family roles, mothers are more likely to live with adult children than fathers (Fry 2016; Kahn et al. 2013; Swartz 2009). Additionally, parent–adult child coresidence is rarer among white compared to African American and Hispanic families (Fry 2016; Keene and Batson 2010; Swartz 2009). These patterns underscore the prospect that living with an adult child is a natural and expected part of the life course for some adults, particularly those in poorer health, those experiencing work or marital-role losses, mothers, and members of ethnic minority groups (Keene and Batson 2010). Transitioning to coresidence with adult children may thus be less distressing for these types of parents than for others.

Additionally, harkening back to the life course focus on social contexts, there are clear geographic patterns in multigenerational coresidence within the United States. Variations in local economies and the availability of housing create different structural constraints on families’ living arrangements (Dong and Hansz 2016; Rappaport 2015; Vespa 2017). Parents and adult children are most likely to coreside in the northeastern states, where housing costs are often at a premium, and then in the less economically well-off southern states, while multigenerational households are comparatively uncommon in the midwestern and somewhat less common in the western states (Vespa 2017). Additionally, western and northeastern states saw steeper increases in coresidence from 2005 through 2015 than did the midwestern states (Vespa 2017). There are also geographic differences in the length and pace of the transition to adulthood that may influence regional norms about coresidence. For instance, transitions to marriage occur earlier in the Midwest and South than in northeastern states, and larger shares of young adults ages 20 to 24 are employed in the Midwest than in other regions (U.S. Bureau of Labor Statistics 2017; U.S. Census Bureau 2017). Assuming that local norms contribute to parents’ attitudes toward coresiding with adult children, these patterns may be reflected in regional variations in the relationship between coresidence and parents’ mental health.

In sum, sociodemographic and regional patterns support these additional hypotheses about variations in the expected negative mental health effects of transitions to coresidence:

**Hypothesis 2:** Transitions to coresidence with adult children will be associated with greater increases in depressive symptoms among parents for whom they are less common (white, male, partnered, working, and nonlimited individuals) than among those for whom they are more likely to be expected (racial-ethnic minority, female, single, retired, and functionally limited parents).
**Hypothesis 3a:** Transitions to coresidence with adult children will be associated with greater increases in depressive symptoms in the Midwest, which has the lowest rates of parent–adult child coresidence, than in the Northeast, where these living arrangements have been more common.
**Hypothesis 3b:** Transitions to coresidence occurring in southern and western states are also predicted to be associated with somewhat greater depressive symptom increases than in the Northeast.

### Contexts of New Parent–Adult Child Coresidence Transitions

The life course principle of timing highlights that transitions and events should be interpreted in the context of when they occur in an individual’s life (Elder 1994; Elder et al. 2003). Although norms are evolving, ideas about when it is and is not appropriate for adult children to live with parents remain widespread (Furstenberg 2010; Settersten et al. 2015; Sharon 2016). Coresidence with adult children may be experienced more negatively when children are older, violating age norms about independence (Settersten 1998). Additionally, evidence suggests that parents most readily support adult children who are the neediest, while those who have achieved other role transitions marking adulthood receive less support (Swartz et al. 2011). It is thus possible that coresiding with children who are unpartnered, childless, or not working is less distressing than living with children who are employed and have their own families because the child’s needs feel justifiable (Settersten 1998). On the other hand, research also indicates that living with adult children who are experiencing work and relationship difficulties can be stressful (Aquilino and Supple 1991; Pearlin et al. 1981; Suitor and Pillemer 1988). Not only may parents take their child’s setbacks personally, but the support needs of nonworking and single children may be particularly taxing emotionally and financially (Fingerman et al. 2012; Gilligan et al. 2018).

Another key emphasis of the life course perspective is that individuals’ lives are inherently interlinked (Bengston and Allen 1993; Elder 1994; Gilligan et al. 2018). Intergenerational coresidence involves family units, and details about relational contexts and motivations for coresiding can provide significant insight to questions about how it might be related to parental mental health. It is now clear that multigenerational households have grown in a large part because families are adapting to meet each other’s needs in a changing economic environment (e.g., Kahn et al. 2013; Maroto 2017; Swartz et al. 2011). Coresidence that occurs because of financial or other personal setbacks encountered by the younger generation may result in these stressors flowing up to the older generation. Need may also be indicated by the type of coresidential move—specifically, whether a child moved back to a parental home or the parent moved to the child’s home. Move direction provides additional context about how coresidential transitions are intended to shift resources within a family, with a returning child potentially indicating the introduction of parental stressors.

Previous research has often dealt with questions about relative need obliquely, looking to the life course transitions of both parties and the health status of parents (Seltzer and Friedman 2014; Smits et al. 2010; Tosi and Grundy 2018; Ward et al. 1992). However, when new household members are reported, the HRS asks which party moved and whose needs the move was intended to fulfill. The only prior study to make use of these questions showed that among unmarried respondents who were 70 and older in 1995, coresidential moves more often occurred to help the parent than the adult child (Choi 2003). It also found that child beneficiaries were more likely to be unemployed and that in cases where the parents were the beneficiary, the child was typically married. These intriguing findings are no doubt influenced by the decision to focus on unmarried respondents and reflective of the earlier cohort and more advanced age of the sample at this time. Updated investigations of these patterns are needed.

Drawing on these possibilities, this article explores the following last hypotheses about variations in mental health among parents with newly coresidential adult children:

**Hypothesis 4:** Assuming that coresidential transitions are less stressful if they involve children for whom they are considered more necessary or normative, parents who move in with children who are married, parents, working, and older may report a greater increase in depressive symptoms than those transitioning to coresidence with children who hold fewer adult social roles and are younger.
**Hypothesis 5a:** Because it may indicate that the adult child encountered stressors that pushed him or her home, coresidential moves in the direction of the parents’ own home will be associated with greater increases in parental depressive symptoms than transitions to an adult child’s home.
**Hypothesis 5b:** Additionally, parents who indicated that the coresidential transition was for their child’s benefit will experience a greater increase in depressive symptoms than parents who named themselves the main beneficiary of the move.

## Methods

### Data and Sample

The HRS (Juster and Suzman 1995) is an ongoing, nationally representative panel survey of U.S. adults age 50 and older. It is supported by the National Institute on Aging and conducted by researchers at the University of Michigan. The study began in 1992 with a sample of adults born between 1931 and 1941, and six cohorts have been added since. To capture the years roughly during and immediately after the Great Recession—the recent time period during which multigenerational households in the United States increased the most sharply (Cohn and Passel 2016)—data for this analysis came from the 2008, 2010, and 2012 waves of the HRS. The sample thus included observations for respondents born before 1953 and their spouses who were also at least 50 in 2010. Most variables were drawn from the individual-level RAND HRS data file, a merged version of the multilevel HRS data designed to increase its accessibility (Bugliari et al. 2016). Additional information about the children of the participants and reports of coresidential moves were merged to the RAND data file from raw HRS files for each year.

The total number of respondents participating in the 2008 survey who were reinterviewed in 2012 was 13,711. After excluding those who reported no living adult children in 2010 (*n*  = 1,106), living in nursing homes in 2008 or 2010 (*n*  = 432) or outside of the four main U.S. Census regions (*n*  = 18), spousal partners under age 50 in 2010 (*n*  = 158), and those missing information on the dependent variable (*n*  = 547), the potential analytic sample consisted of 11,468 individuals. Missing data on another 191 cases were dealt with by listwise deletion, resulting in an analytic sample size of 11,277, or 98.3% of the potential sample after exclusions. Most missing information was on depressive symptoms in 2008 (*n*  = 146). Supplemental analyses multiply imputing missing data for these 191 cases produced substantively identical findings to those using listwise deletion.

### Measures

#### Adult child coresidence transitions

Respondents provided detailed information about household members in all waves. Based on the identification of new and preexisting household members ages 18 and older who were children or stepchildren, the following dummy variables for adult children’s coresidence transitions between 2008 and 2010 were created: stably without coresidential adult children, stably coresidential adult child, exit of coresidential adult child, and new coresidential adult child. The columns of Table 1 show sample sizes for each of these groups, along with descriptive statistics and bivariate analyses. Of the 11,277 parents of adult children that comprised the analytic sample, over 75% (*n* = 8,487) reported no coresidential adult children in 2008 or 2010. Another 15.7% indicated that the same adult child lived in their household at both times (*n* = 1,760), and about 7.5% reported that a coresident adult child in 2008 lived elsewhere by 2010 (*n* = 841). Just 4.4% of respondents (*n* = 520) reported that an adult child who did not live with them in 2008 was a coresident in 2010. There was no overlap between those stably without coresidential children and the other adult child coresidence categories. However, 168 of the respondents with a stably coresidential adult child also reported the exit of a coresidential adult child between 2008 and 2010, 97 respondents reported both a stably and newly coresidential adult child, and 60 both lost and gained a coresidential adult child.

**Table 1.** Means (and Standard Deviations) of Study Variables by Adult Child Coresidential Transitions, 2008 to 2010.

Variable	Total (*N =* 11,277)	Stably without coresidential adult children (*n* = 8,476)	Stably coresidential adult child (*n* = 1,760)	Exit of coresidential adult child (*n* = 841)	New coresidential adult child (*n* = 520)
Depressive symptoms 2012 (0–8)	1.39 (1.94)	1.31 (1.88)	1.58* (2.02)	1.47* (2.07)	1.85* (2.16)
Depressive symptoms 2008 (0–8)	1.34 (1.91)	1.25 (1.84)	1.54* (2.00)	1.64* (2.17)	1.68* (2.16)
Functional limitations 2008 (0–5)	.20 (.68)	.19 (.63)	.28* (.81)	.22 (.73)	.32* (.86)
Female (%)	60.67	58.95	66.19*	63.61*	69.62*
Age 2010 (50–101)	69.87 (9.19)	70.72 (8.91)	67.69* (9.72)	64.75* (8.63)	68.49* (9.51)
U.S. census region (%)					
Northeast	14.37	14.07	16.48*	14.51	11.35
Midwest	25.92	27.11	21.53*	25.80	19.62*
South	40.49	40.11	40.45	42.21	42.12
West	19.21	18.71	21.53*	17.48	26.92*
Race-ethnicity (%)					
White	74.30	79.72	54.66*	60.88*	55.00*
African American	13.99	11.82	20.85*	20.33*	23.46*
Hispanic	9.45	6.45	21.03*	16.88*	18.27*
Other	2.26	2.01	3.47*	1.90	3.27*
Years of education (0–17)	12.66 (3.12)	12.88 (2.91)	11.79* (3.67)	12.34* (3.69)	11.73* (3.54)
Household income 2010 (%)					
1st quartile	25.00	22.41	34.43*	28.06*	37.69*
2nd quartile	25.00	25.69	22.39*	22.12*	26.15
3rd quartile	25.00	26.35	20.74*	21.88*	17.50*
4th quartile	25.00	25.54	22.44*	27.94	18.65*
Employed 2010 (%)	32.34	30.01	38.87*	46.86*	33.46
Marital status 2010 (%)					
Married/partnered	66.30	68.90	58.75*	64.80*	47.69*
Separated/divorced	11.70	10.71	13.47*	15.22*	17.31*
Widowed	20.72	19.18	26.12*	18.74	32.70*
Never married	1.28	1.19	1.65	1.19	2.12
Number of adult children (1–20)	3.53 (2.08)	3.40 (2.00)	3.90* (2.19)	4.08* (2.39)	4.40* (2.40)

* Significantly different from those stably without coresidential adult children at *p < .*05, two-tailed *t* tests.

#### Depressive symptoms

Depressive symptoms in 2012 was based on nine items from the Center for Epidemiologic Studies Depression Scale, a survey instrument that assesses depressive symptoms with high reliability and validity (Radloff 1977). Participants reported whether in the past week they had (1) felt that everything was an effort, (2) experienced restless sleep, (3) felt happy, (4) felt lonely, (5) enjoyed life, (6) felt sad, (7) felt that they could not get going, and (8) had a lot of energy. Items 3 and 8 were reverse coded and responses were summed, resulting in a scale that ranged from 0 to 8. The Cronbach’s alpha for the items was .81. Table 1 shows that the average depressive symptoms score in 2012 was 1.39. The bivariate analyses indicate that respondents with a coresidential adult child at either time point reported higher depressive symptoms scores in 2012 than those stably without coresidential children. Depressive symptoms in 2008 was measured with the same items as in 2012 and also ranged from 0 to 8. The mean score on depressive symptoms in 2008 was 1.34 (Table 1).

#### Functional limitations

To account for prior physical health the analyses control for functional limitations in 2008. This measure was composed of five items asking whether the respondent experienced difficulty walking across a room, getting dressed, bathing, eating, getting in and out of bed, and using the toilet, and ranged from 0 to 5. The mean for this item was .20. Respondents with a stably or newly coresidential adult child reported higher depressive symptom and functional limitation scores in 2008 than did parents stably living on their own.

#### Demographic and status characteristics

Gender was measured with a dummy variable for female. Geographic regions, as defined by the U.S. Census, included the Northeast, Midwest, West, and South (U.S. Census n.d.). Age in 2010 was measured continuously from 50 to 101 years of age, and race-ethnicity was measured with a series of dummy variables for white, African American, Hispanic, and a heterogeneous *other* category. Years of education was measured continuously from 0 to 17. Household income in 2010 was measured by quartiles based on the continuous dollar amount of 2010 household income, for which missing values were imputed in several stages by RAND scholars (for details, see Bugliari et al. 2016). Work in 2010 was also binary and indicated whether the respondent reported paid work. Marital status in 2010 consisted of dummy variables for married/partnered, separated/divorced, widowed, and never married. The analyses also controlled for the number of adult children reported by each respondent in 2010, which was directly related to the odds of having a coresidential child. Sample means and proportions for all of these variables are again shown in the first column of Table 1. The bivariate analyses show that more respondents with coresidential adult children at either point were female, living in the Northeast or West, racial-ethnic minorities, separated or widowed, in the bottom income quartile, and employed, while fewer were white, in the top income quartile, or married. Those reporting a coresidential adult child were also older, completed fewer years of schooling, and had more adult children on average than those stably without adult child coresidents. Respondents reporting the exit of a coresidential child tended to be more similar to those stably without coresidential children.

#### Type of coresidential move

HRS respondents claiming a new household member were asked whether they moved into this person’s home or the new member moved in with them. Based on responses to this question, the 520 individuals reporting a new adult child household member in 2010 were grouped into the following categories: child moved to parental home, parent moved to child’s home, and other type of move, which combined those who answered that both parties moved and those specifying some other situation. The number of respondents reporting each type of move is shown across the columns of Table 2. Over 75% (*n*  = 392) reported that an adult child moved into their home, about 16% (*n* = 83) answered that they moved to a child’s home, and 10% (*n* = 52) reported that both parties moved or that some other circumstances surrounded the move. Note that because a small number (*n* = 28) of respondents reported more than one newly coresidential adult child, these categories were not constrained to be mutually exclusive. Bivariate analyses in Table 2 indicate that respondents who moved to their child’s home between 2008 and 2010 were more depressed in 2008, older, more likely to be Hispanic, lower income, and less likely to be employed, and reported fewer years of education than respondents whose child moved to their home. Additionally, those who moved into a child’s home were less likely to be married or partnered and more likely to be widowed or divorced than those with a child who moved to their home.

**Table 2.** Means (and Standard Deviations) of Study Variables by Type of Move among Parents with a New Coresidential Adult Child in 2010 (*N* = 520).

Variable	Child moved to parental home (*n* = 392)	Parent moved to child’s home (*n* = 83)	Other type of move (*n* = 52)
Depressive symptoms 2012 (0–8)	1.79 (2.19)	2.16 (2.05)	1.85 (2.24)
Depressive symptoms 2008 (0–8)	1.58 (2.08)	2.13* (2.32)	1.77 (2.47)
Functional limitations 2008 (0–5)	.29 (.87)	.45 (.80)	.37 (1.03)
Female (%)	68.62	75.90	65.38
Age 2010 (50–96)	67.19 (8.92)	74.30* (10.60)	69.04 (8.60)
U.S. census region (%)			
Northeast	11.22	15.66	3.85
Midwest	21.43	13.25	13.46
South	42.09	44.58	36.54
West	25.26	26.51	46.15*
Race-ethnicity (%)			
White	56.38	49.40	51.92
African American	23.98	16.87	26.92
Hispanic	16.84	28.92*	17.31
Other	2.81	4.82	3.85
Years of education (0–17)	11.84 (3.45)	10.64* (3.85)	12.62 (3.38)
Household income 2010 (%)			
1st quartile	31.63	69.88*	30.77
2nd quartile	27.55	20.48	30.77
3rd quartile	19.13	4.82*	23.08
4th quartile	21.68	4.82*	15.38
Employed 2010 (%)	39.54	9.64*	23.08*
Marital status 2010 (%)			
Married/partnered	54.59	13.25*	50.00
Separated/divorced	15.31	24.10	21.15
Widowed	28.32	59.04*	26.92
Never married	1.79	3.61	1.92
Number of adult children 2010 (1–14)	4.33 (2.36)	4.48 (2.43)	4.60 (2.64)
Characteristics of new coresidential child(ren) (%)			
Age 35+	59.95	81.93*	80.77*
Not employed	42.86	31.33	34.62
Not partnered	80.61	37.35*	21.15*
Nonparent	40.05	19.28*	28.85
Beneficiary of coresidential move (%)			
Child	60.46	2.41*	50.00
Parent	3.57	45.78*	13.46*
Both	36.48	49.40*	32.69
Other	1.79	2.41	5.77*

* Significantly different from parents with children who moved to their home at *p* *< .*05, two-tailed *t* tests.

#### Beneficiary of coresidential move

Respondents with a new household member were also asked which party the move was intended to help. Based on answers to this question, four groups were created: move helped child, move helped parent, move helped both, and other type of benefit. Cell sizes for these groups are shown across the columns of Supplemental Table 1 in the online version of the article, along with additional descriptive characteristics. Over 50% (*n*  = 262) of those with a newly coresidential adult child answered that the move occurred primarily to benefit the child, 11.2% reported that it benefited them (*n*  = 58), 38.1% (*n*  = 198) answered that the move was beneficial for both parties, and just 2.1% (*n*  = 11) gave some other answer.

#### Characteristics of newly coresidential adult children

Finally, several characteristics of newly coresidential adult children are examined. This information comes from the raw HRS household-level files that provide details about every member of the household. Dummy variables indicated the presence of newly coresidential children who were age 35+ (vs. 18–34), not employed, not partnered, and a nonparent in 2010. Table 2 shows that most parents who moved into a child’s home or experienced another type of move reported that the child was at least 34 years old, but fewer than 60% of parents who had a child return to their home indicated that the child was 35+. A smaller proportion of respondents who moved to a child’s home identified the other party as not working or being childless. Compared to those whose child moved to their home, fewer respondents in both other groups indicated that their newly coresidential child was partnered. A majority of those whose adult child moved into their home reported that the move was for the child’s benefit (over 60%), compared to just 2.41% of those moving into a child’s home. Fewer than 4% of respondents whose child moved to their home said it was for their own benefit, compared to nearly 50% of respondents moving to their child’s home.

### Analytic Strategy

The analysis proceeded in three stages. First, respondents’ depressive symptoms in 2012 were regressed on adult child coresidential transitions from 2008 through 2010 using ordinary least squares (OLS) regression (Table 3). These analyses show whether the later depressive symptoms scores of respondents with a stably coresidential adult child, who moved apart from a coresidential adult child, or with a newly coresidential adult child in 2010 differed from those of their community-dwelling counterparts without coresidential adult children in 2008 through 2010. Controls for earlier depressive symptoms were included in Model 1, and other sociodemographic characteristics of parents were added in Model 2. Model 3 shows the significant interactions between covariates and having newly coresidential children, which reveal whether the mental health effects of these transitions varied across parental characteristics. Since all models included prior depressive symptoms, coefficients are interpretable as changes in depressive symptoms over time.

**Table 3.** Coefficients from Ordinary Least Squares Regression of Depressive Symptoms in 2012 on Adult Child Coresidential Transitions, 2008 to 2010 (*N =* 11,277).

Variable (reference)	Model 1	Model 2	Model 3
Depressive symptoms 2008	.540*** (.008)	.468*** (.012)	.468*** (.012)
Adult child coresidential transitions (no coresidential adult children)			
Stably coresidential adult child	.103* (.044)	.026 (.045)	−.191 (.099)
Exit of coresidential adult child	−.108 (.063)	−.090 (.063)	−.071 (.187)
New coresidential adult child	.285*** (.084)	.179* (.082)	−.299 (.185)
Functional limitations 2008		.282*** (.031)	.281*** (.032)
Female		.065* (.031)	.065* (.031)
Age 2010		−.005* (.002)	−.005* (.002)
U.S. census region (Northeast)			
Midwest		−.078 (.050)	−.137* (.057)
South		.045 (.048)	−.001 (.055)
West		.063 (.055)	.014 (.062)
Race-ethnicity (white)			
African American		−.014 (.049)	−.014 (.049)
Hispanic		−.017 (.069)	−.020 (.070)
Other		.110 (.103)	.098 (.103)
Years of education		−.038*** (.006)	−.038*** (.006)
Household income quartile 2010		−.099*** (.019)	−.099*** (.019)
Employed 2010		−.207*** (.037)	−.207*** (.037)
Marital status 2010 (married)			
Separated/divorced		.186*** (.058)	.189*** (.059)
Widowed		.067 (.048)	.070 (.048)
Never married		−.206 (.139)	−.202 (.139)
Number of adult children		−.009 (.008)	−.009 (.008)
Stably coresidential child × Midwest			.235 (.132)
Stably coresidential child × South			.231 (.120)
Stably coresidential child × West			.345* (.144)
Exit of coresidential child × Midwest			−.101 (.218)
Exit of coresidential child × South			−.151 (.211)
Exit of coresidential child × West			−.415 (.232)
New coresidential child × Midwest			.708** (.263)
New coresidential child × South			.481* (.223)
New coresidential child × West			.496* (.250)
*R* ^2^	.286	.314	.315

*Note*: Standard errors in parentheses.

* *p* < .05, **p < .01, ***p < .001.

Next, analyses in Table 3 were repeated with the use of a weight for the inverse probability of having a new coresidential adult child in 2010 (Table 4). As described previously, existing research shows that a number of characteristics of adults increase the likelihood that they experience a transition to coresidence with an adult child. Inverse-probability weighting helps adjust for this nonrandom selection into two different treatment groups (Austin 2011). To create the probability weights, a logistic regressions was estimated for the presence of a new coresidential adult child in 2010 on depressive symptoms, functional limitations, age, income, work status, and marital status in 2008, as well as gender, region, race-ethnicity, and years of education, using the respondent-level survey weight for 2010 calculated by HRS researchers. With *p* as the probability of having new coresidential adult children, respondents who did report them received a weight of $\frac{1}{p}$, while those who did not have new coresidential adult children in 2010 received a weight of $\frac{1}{1-p}$.

**Table 4.** Coefficients from Inverse-Probability Weighted Ordinary Least Squares Regression of Depressive Symptoms in 2012 on Adult Child Coresidential Transitions, 2008 to 2010 (*N* = 11,277).

Variable (reference)	Model 1	Model 2
Depressive symptoms 2008	.474*** (.028)	.474*** (.029)
Adult child coresidential transitions (no coresidential adult children)		
Stably coresidential adult child	.010 (.113)	.014 (.114)
Exit of coresidential adult child	−.067 (.161)	−.074 (.163)
New coresidential adult child	.183* (.082)	−.255 (.211)
U.S. census region (Northeast)		
Midwest	.128 (.124)	−.116 (.071)
South	.266* (.119)	−.022 (.051)
West	.262 (.154)	.067 (.087)
Stably coresidential child × Midwest		.228 (.243)
Stably coresidential child × South		.481 (.258)
Stably coresidential child × West		.136 (.285)
Exit of coresidential child × Midwest		−.407 (.413)
Exit of coresidential child × South		−.417 (.433)
Exit of coresidential child × West		−.784 (.503)
New coresidential child × Midwest		.500 (.267)
New coresidential child × South		.517* (.247)
New coresidential child × West		.486 (.292)

*Note:* Standard errors in parentheses. All models also control for depressive symptoms and functional limitations in 2008, gender, age in 2010, race-ethnicity, education, income, work status, marital status, and number of adult children in 2010.

* *p* < .05, **p < .01, ***p < .001.

Finally, Table 5 shows variations in mental health among respondents with adult children who returned to their home based on the characteristics of the coresidential child, including their age (Model 1), employment status (Model 2), partnership status (Model 3), and parental status (Model 4). The last two models explore how the type of coresidential move (Model 5) and the parents’ account of the main beneficiary of the move (Model 6) were related to later depressive symptoms. To account for nonindependence in households in which both a primary respondent and a spouse contributed responses, standard errors were adjusted for clustering at the household level.

**Table 5.** Coefficients from Ordinary Least Squares Regressions of Depressive Symptoms in 2012 on Coresidential Adult Child and Coresidential Move Characteristics among Parents with Newly Coresidential Children from 2008 to 2010 (*N* = 520).

Variable (reference)	Model 1	Model 2	Model 3	Model 4	Model 5	Model 6
Age 35+ (ages 18–34)	.042 (.224)					
Not employed (employed)		.522** (.178)				
Not partnered (partnered)			−.178 (.189)			
Nonparent (parent)				−.270 (.186)		
Type of coresidential move (child moved)						
Parent moved					−.180 (.253)	
Both moved/other type of move					−.117 (.275)	
Main beneficiary of coresidential move (child)						
Parent						.100(.314)
Both						−.049 (.195)
Other						.478 (.506)

*Note:* All models control for depressive symptoms and functional limitations in 2008, gender, age in 2010, region, race-ethnicity, education, income, work status, marital status, and number of adult children in 2010.

* *p* < .05, **p < .01, ***p < .001.

## Results

The first set of multivariate results in Table 3 shows coefficients from OLS regressions of depressive symptoms in 2012 on 2008 to 2010 adult child coresidential transitions and covariates. Model 1 tests Hypothesis 1—that parents who transitioned to coresidence with an adult child would experience an increase in depressive symptoms relative to parents who did not live with a child during this period, while those stably living with or experiencing the exit of an adult child would not differ from independent parents in terms of their depressive symptoms. The model first shows that depressive symptoms in 2008 were robust positive predictors of depressive symptoms in 2012. Then, compared to those who had no coresidential adult children from 2008 to 2010, both parents with adult children who were stable or new coresidents reported an increase in depressive symptoms in 2012. However, the relationship between having stably coresidential child and depressive symptoms disappears with the addition of covariates in Model 2. Although the relationship is also reduced, respondents claiming a newly coresidential adult child in 2010 still reported a greater increase in depressive symptoms scores than those stably without children after accounting for sociodemographic differences in Model 2. Specifically, those with a newly coresidential adult child between 2008 and 2010 reported later depressive symptom scores that were .179 points higher than those of respondents without coresidential adult children at this time. This is greater than the increase in depressive symptoms associated with being female (.065) and close to (if less significant than) the increase associated with being separated or divorced in 2010 (.186). Together, these findings provide support for Hypothesis 1. Model 2 also shows that increases in depressive symptoms scores from 2008 to 2010 were positively associated with functional limitations scores in 2008. Female, nonworking, and separated or divorced respondents reported an increase in depressive symptoms scores relative to male, employed, and married respondents. Depressive symptoms scores decreased as age, years of education, and income quartile increased.

Analyses interacting each of the covariates (including a categorical measure of age distinguishing between respondents 50 to 64 and 65+) with the coresidence dummies revealed only one significant set of results, shown in Model 3: compared to parents located in the northeastern states, new coresidential children resulted in a greater increase in depressive symptoms for parents in all other geographic regions. Additionally, parents with a stably coresidential child reported a greater increase in depressive symptoms if they were in the West than in the Northeast. The results of interaction analyses thus provide no support for Hypothesis 2—that transitions to coresidence would predict greater increases in depressive symptoms among those for whom they are less common and may have been more unexpected, including white, male, partnered, working, and nonlimited parents. However, they appear to support Hypothesis 3 about regional variations, showing that parents who moved in with adult children in the Midwest, South, and West experienced greater increases in depressive symptoms than those in the Northeast. More specifically, having newly coresidential children was associated with increases in depressive symptoms scores that were higher by .708 points in the Midwest, .481 points in the South, and .496 points in the West than in the Northeast.

Table 4 repeats analyses from Models 2 and 3 in Table 3 with the addition of inverse-probability weights that adjust for nonrandom selection into having newly coresidential adult children in 2010 based on 2008 health and characteristics. While having a newly coresidential child still predicts an increase in parents’ depressive symptoms, the only lingering geographic difference in this effect is that parents living in the South were more depressed in association with these moves than those living in the northeastern states. Hence, Hypothesis 3, about variations in the effects of coresidence transitions by geographic region, is also largely unsupported after this adjustment. In other words, while transitions to coresidence that occurred in the West and Midwest were associated with an increase in later depressive symptoms relative to those occurring in the Northeast, these differences are explained by regional variations in the types of parents that select into coresidential transitions. Supplemental analyses using an inverse-probability-weighted regression adjustment that matches treated respondents (i.e., those with newly coresidential adult children) to three nearest neighbors (*teffects ipwra* in Stata 15) show that the average effect of having a new coresidential child on depressive symptoms scores is an increase of .219 (*p* < .05).

The last set of analyses in Table 5 restricts the sample to the 520 respondents reporting that they had a newly coresidential adult child between 2008 and 2010 to assess Hypotheses 4 and 5 about whether characteristics of the adult child, the direction of the move, or the reported beneficiary is related to changes in depressive symptoms in this group. Whereas the child’s age, partnership, and parental status are all unrelated to the depressive symptoms of parents with newly coresidential adult children, transitioning to coresidence with an out-of-work child was associated with a greater increase in depressive symptoms than transitions involving an employed adult child. That is, parents who transitioned to coresidence with an adult child who was not working reported an increase in depressive symptoms scores that was .522 points higher relative to parents whose transition involved a working child. Although the coefficients for controls are not shown, this increase is again comparable to the increase associated with experiencing a marital loss in this subgroup. Analyses in Supplemental Table 2 (in the online version of the article) that focus on parents without coresidential adult children suggest that results in Table 5 do not reflect that simply having any children who are struggling with employment was depressing; having one or more noncoresidential unemployed children was not related to parents’ depressive symptoms. This one significant finding runs counter to Hypothesis 4, that coresidence transitions involving children who achieved more adult social roles might be more depressing since they are interpreted as less needy or deserving. Models 5 and 6 of Table 5 indicate that the direction of the move and parents’ reports of whom the child’s return was intended to help were unrelated to depressive symptoms, also providing no support for Hypothesis 5, that moves indicating child’s need would predict the greatest increases in distress.

## Discussion

The central finding of this article is that parents ages 50 and older with newly coresidential adult children between 2008 and 2010 experienced an increase in depressive symptoms relative to their peers without coresidential children during this period. Parents who experienced a transition to coresidence with adult children were also mentally and physically less healthy in 2008 than those who did not, resonating with research showing that such moves can occur as a result of parents’ health declines and needs for support (Seltzer and Friedman 2014; Smits et al. 2010). However, even after accounting for health and other factors selecting parents into coresidence transitions, respondents with new coresidential adult children reported more depressive symptoms two years after. Additionally, the analyses show that the change in depressive symptoms for parents with stably coresidential adult children, or who experienced the departure of a coresidential child, did not differ from that of respondents living apart from their adult children. The life course principle of timing provides useful tools for interpreting these findings (Elder et al. 2003), which suggest that the relationship between coresidence with adult children and parents’ mental health was dependent on whether the arrangement was new or longer term. There may be something particular to the experience of returning to a coresidential arrangement after a period of living independently from adult children that can be distressing for parents. In addition to the introduction of new stressors that require an adjustment period, having a newly coresidential child may be distressing because it represents the loss of a positively anticipated empty-nest stage (Aquilino and Supple 1991; Barber 1989). After parents have adapted to the household changes, these changes may no longer take a toll on well-being. Supplemental analyses (available on request) support the perspective that the negative effects of transitions to coresidence with children are relatively short-lived; by 2014, the relationship between 2008 and 2010 coresidence transitions and depressive symptoms was not significant.

Following the life course principle of time and place, these main findings should be interpreted within the context of when and where they occurred. The period from 2008 to 2010 was marked by major economic setbacks in the United States. One likely reason why contemporary transitions to coresidence with adult children were associated with parents’ depressive symptoms during this period is because they arose from and created economic strains (Mykyta and Macartney 2011). Supplemental analyses exploring the relationship between transitions to coresidence with adult children experienced from 2006 to 2008 and parental mental health from 2006 to 2010 (available by request) show that parents with newly coresidential children at this earlier time point did not experience a similar increase in depressive symptoms. This highlights the possibility that the transitions examined here were distressing because they accompanied (and resulted from) an economic crisis. Cross-cohort and cross-period comparisons of multigenerational household transitions, as well as of the relationship between adult children’s circumstances and their parents’ well-being more broadly, are a promising and important direction for further research.

National context is also important. The finding that parents with newly coresidential children experienced increases in depression is consistent with an earlier U.S. study suggesting that living with young adult children is distressing for parents (Pudrovska 2009) but at odds with much of the recent research based in other nations (e.g., Aranda 2015; Chen and Short 2008; Courtin and Avendano 2016; Grundy and Murphy 2017). Broad cross-national variations in the historical background and meaning of parent–child coresidence may contribute to these inconsistences, with coresidential living arrangements potentially being more distressing where they are less normative. Supporting this possibility, a recent study (Tosi and Gundy 2018) indicates that adult children’s returns to the parental nest resulted in greater decreases in parents’ quality of life in Nordic countries—which have strong welfare states that have historically supported young adults’ independence—than in other European countries. Evidence indicates that self-sufficiency is one of the values Americans hold most dear (Andreß and Heien 2001; Linos and West 2003), and most U.S. adults continue to believe that residential independence is an important marker of a successful transition to adulthood (Furstenberg 2010; Sharon 2016). Additionally, qualitative research indicates that having coresidential adult children is disappointing for at least some American parents (Newman 2012). In short, a transition to coresidence with adult children may both be financially stressful and violate the values and expectations of parents in the contemporary United States.

The analyses in this article reveal little variation in the relationship between having a newly coresidential adult child and parental mental health. Inconsistent with expectations, parental characteristics did not condition the effects of coresidence transitions on parental depression. Additionally, while transitions occurring in the southern United States were associated with a greater increase in depressive symptoms than those occurring in the northeastern states, there were no differences for the midwestern and northwestern states, despite the Midwest being the region where multigenerational households are by far the rarest (Vespa 2017). The findings thus do not support the hypothesis that moving back in with an adult child is more distressing among those for whom it may be the least expected, and suggest that the modest depressing effect applied across groups. One potential explanation for the finding that transitioning to coresidence with adult children was associated with more depressive symptoms in the South is that this is the most economically deprived U.S. region (Semega, Fontenot, and Kollar 2018), where the financial strains associated with these moves may have been especially profound.

Also largely at odds with the hypotheses, analyses focusing on parents with newly coresidential adult children indicate that child characteristics were generally unrelated to parental mental health. Only work status mattered, again pointing to the probable role of economic stress in explaining why having a newly coresidential adult child is distressing. This finding resonates with the life course focus on linked lives, which highlights how both resources and stressors are transmitted through members of a family (Bengston and Allen 1993). To the extent that multigenerational coresidence has increased because families are adapting to an economic context that has been particularly unfavorable to the younger generation (Bell and Blanchflower 2011), it represents a pathway through which the stressors encountered by one generation can be absorbed by and impact the other. However, unexpectedly, neither the direction of the coresidential move nor parents’ reports of the primary beneficiary of the move seemed to matter for parental depression. While this is likely driven at least in part by small cell sizes, and there is no way to assess the accuracy of parents’ reports of beneficiaries, analyses based on larger samples of adults experiencing a return to coresidence with adult children are needed.

In addition to the relatively small number of respondents who experienced a transition to coresidence with an adult child, these analyses are limited in their ability to provide a definitive answer to why these transitions appear to be distressing for parents. Although findings suggesting that transitions to coresidence involving out-of-work children are particularly depressing offer preliminary support for the possibility that economic demands are a driver, more research is needed. Specifically, future research should explore the possible mechanisms more directly by making use of multiwave measures of financial and emotional strain and attitudes toward multigenerational households. Unfortunately, the HRS does not include this information.

Even with these limitations, these analyses provide timely insight to the question of how recent and ongoing increases in multigenerational coresidence in the United States may be affecting the well-being of aging parents. Specifically, the results suggest that moving back in with adult children can be depressing and that parents with out-of-work return coresidential children may be particularly vulnerable to increases in depressive symptoms. While the presence of longer-term coresidential children did not predict parental depression, there is no case in which having a coresidential adult child improved parents’ mental health. Insights from the life course perspective sensitize us to the importance of the U.S. context and life course timing when interpreting these transitions. More broadly, the findings highlight that one of the most important ways that U.S. families are adapting to a changing economic context—by sharing the same roof—may also result in the sharing and redistribution of certain stressors.

## Supplemental Material

849113_supp_mat – Supplemental material for Crowded Nests: Parent–Adult Child Coresidence Transitions and Parental Mental Health Following the Great Recession Click here for additional data file. Supplemental material, 849113_supp_mat for Crowded Nests: Parent–Adult Child Coresidence Transitions and Parental Mental Health Following the Great Recession by Jennifer Caputo in Journal of Health and Social Behavior
