# Supplementary material for: Crowded Nests: Parent–Adult Child Coresidence Transitions and Parental Mental Health Following the Great Recession
Source: J Health Soc Behav. 2019 May 23;60(2):204–21. doi: 10.1177/0022146519849113 (PMC6573002; doi:10.1177/0022146519849113)
Supplement: 849113_supp_mat – Supplemental material for Crowded Nests: Parent–Adult Child Coresidence Transitions and Parental Mental Health Following the Great Recession [file 849113_supp_mat.pdf]

Supplemental Table 1. Means (and Standard Deviations) of Study Variables by Beneficiary of Move among Parents with a New Coresident Adult Child in the 2010 Wave of the Health and Retirement Study (*N* = 523)

	Helped child (*N* = 262)	Helped parent (*N* = 58)	Helped both (*N* = 198)	Other (*N* = 11)
Depressive symptoms 2012 (0–8)	1.65	2.50*	1.96	2.18
	(2.12)	(2.22)	(2.19)	(2.79)
Depressive symptoms 2008 (0–8)	1.45	2.40*	1.81	1.82
	(2.09)	(2.37)	(2.16)	(2.75)
Functional limitations 2008 (0–5)	.24	.62*	.35	.18
	(.76)	(1.04)	(.93)	(.40)
Female (%)	64.89	74.14	75.25*	63.64
Age 2010 (38–96)	66.13	75.64*	69.28*	72.18*
	(7.83)	(10.30)	(10.19)	(8.82)
U.S. census region (%)				
Northeast	9.54	13.79	13.13	18.18
Midwest	25.19	12.07*	13.64*	18.18
South	40.08	46.55	44.95	36.36

West	25.19	27.59	28.28	27.27
Race-ethnicity (%)				
White	62.60	51.72	45.45*	45.45
African American	19.85	20.69	28.79*	45.45*
Hispanic	16.79	20.69	20.20	9.09
Other	.76	6.90*	5.56*	.00
Years of education (0–17)	12.23	10.97*	11.11*	13.55
	(3.26)	(3.92)	(3.66)	(3.33)
Household income 2010 (%)				
1st quartile	22.52	67.28*	50.51*	18.18
2nd quartile	25.57	22.41	28.28	36.36
3rd quartile	22.90	5.17*	14.14*	9.09
4th quartile	29.01	5.17*	7.07*	36.36
Employed 2010 (%)	40.84	8.62*	29.80*	27.27
Marital status 2010 (%)				
Married/partnered	66.79	18.97*	29.80*	45.45
Separated/divorced	13.36	25.86*	20.71*	18.18

Widowed	19.08	53.45*	45.96*	27.27
Never married	.76	1.72	3.54*	9.09*
Number of adult children 2010 (1–14)	4.11	4.90*	4.64*	4.09
	(2.26)	(2.70)	(2.50)	(1.70)
Characteristics of new coresidential child(ren) (%)				
Age 35+	60.69	86.21*	65.15	81.82
Not employed	41.22	39.66	38.38	45.45
Not partnered	70.23	46.55*	72.22	63.64
Nonparent	39.31	27.59	32.32	36.36
Type of coresidential move (%)				
Child moved	90.46	24.14*	72.50*	63.64*
Parent moved	.76	65.52*	20.71*	18.18*
Both/other type of move	9.92	12.07	8.59	27.27

* Significantly different from those reporting the move benefited their child $p < .05$, two-tailed tests.

Supplemental Table 2. Coefficients from Ordinary Least Squares Regressions of Depressive Symptoms in 2012 on Adult Child Characteristics among Parents without Coresidential Adult Children 2009–2010 (*N* = 8.476)

	Model1	Model 2	Model 3	Model 4
1+ child age 35+ *(all children age 18–34)*	.016			
	(.022)			
1+ child not employed *(all children employed)*		.048		
		(.038)		
1+ child not partnered *(all children partnered)*			.089*	
			(.038)	
1+ child nonparent *(all children parents)*				–.004
				(.036)

Note. All models control for depressive symptoms and functional limitations in 2008, gender, age in 2010, race-ethnicity, education, and income, work status, marital status, and number of adult children in 2010.

***$p < .001$, **$p < .01$, *$p < .05$
